# Kidney injury in patients with heart failure‐related cardiogenic shock: Results from an international, multicentre cohort study

**DOI:** 10.1002/ejhf.3701

**Published:** 2025-05-28

**Authors:** Jonas Sundermeyer, Caroline Kellner, Benedikt N. Beer, Lisa Besch, Angela Dettling, Letizia Fausta Bertoldi, Stefan Blankenberg, Jeroen Dauw, Dennis Eckner, Ingo Eitel, Tobias Graf, Patrick Horn, Joanna Jozwiak‐Nozdrzykowska, Paulus Kirchhof, Stefan Kluge, Axel Linke, Ulf Landmesser, Enzo Lüsebrink, Nicolas Majunke, Norman Mangner, Sven Möbius Winkler, Peter Nordbeck, Martin Orban, Federico Pappalardo, Matthias Pauschinger, Michal Pazdernik, Alastair Proudfoot, Matthew Kelham, Tienush Rassaf, Hermann Reichenspurner, Clemens Scherer, P. Christian Schulze, Robert H.G. Schwinger, Carsten Skurk, Marek Sramko, Guido Tavazzi, Holger Thiele, Luca Villanova, Nuccia Morici, Ephraim B. Winzer, Dirk Westermann, Benedikt Schrage

**Affiliations:** ^1^ Department of Cardiology University Heart and Vascular Center Hamburg, University Medical Center Hamburg‐Eppendorf Hamburg Germany; ^2^ German Center for Cardiovascular Research (DZHK), Partner site Hamburg/Kiel/Lübeck Hamburg Germany; ^3^ Center for Population Health Innovation (POINT), University Heart and Vascular Center Hamburg, University Medical Center Hamburg‐Eppendorf Hamburg Germany; ^4^ Cardio Center, Humanitas Clinical and Research Center ‐ IRCCS Rozzano Italy; ^5^ Cardiovascular Center Aalst, OLV Hospital Aalst Belgium; ^6^ Department of Cardiology Paracelsus Medical University Nürnberg Nürnberg Germany; ^7^ University Heart Center Lübeck, University Hospital Schleswig‐Holstein Lübeck Germany; ^8^ Division of Cardiology, Pulmonology and Vascular Medicine Medical Faculty, University Duesseldorf Duesseldorf Germany; ^9^ Department of Internal Medicine and Cardiology Heart Center Leipzig at University of Leipzig and Leipzig Heart Science Leipzig Germany; ^10^ Department of Intensive Care Medicine University Medical Center Hamburg‐Eppendorf Hamburg Germany; ^11^ Department for Internal Medicine and Cardiology Heart Center Dresden, Dresden University of Technology Dresden Germany; ^12^ Department of Cardiology, Angiology and Intensive Care Medicine Deutsches Herzzentrum der Charité (DHZC), Campus Benjamin Franklin Berlin Germany; ^13^ Department of Medicine I University Hospital, LMU Munich Munich Germany; ^14^ Department of Internal Medicine I University Hospital Jena Jena Germany; ^15^ Department of Internal Medicine I University Hospital Würzburg Würzburg Germany; ^16^ Department of Cardiothoracic and Vascular Anesthesia and Intensive Care AO SS. Antonio e Biagio e Cesare Arrigo Alessandria Italy; ^17^ Department of Cardiology IKEM Prague Czech Republic; ^18^ Department of Perioperative Medicine St. Bartholomew's Hospital London UK; ^19^ Department of Cardiology and Vascular Medicine West German Heart and Vascular Center, University Hospital Essen Essen Germany; ^20^ Department of Cardiothoracic Surgery University Heart and Vascular Center Hamburg, University Medical Center Hamburg‐Eppendorf Hamburg Germany; ^21^ Medizinische Klinik II, Kliniken Nordoberpfalz AG Weiden Germany; ^22^ Department of Clinical‐Surgical, Diagnostic and Paediatric Sciences University of Pavia Pavia Italy; ^23^ Anesthesia and Intensive Care, Fondazione Policlinico San Matteo Hospital IRCCS Pavia Italy; ^24^ Unità di Cure Intensive Cardiologiche and De Gasperis Cardio‐Center ASST Grande Ospedale Metropolitano Niguarda Milan Italy; ^25^ IRCCS Fondazione Don Gnocchi, ONLUS, Santa Maria Nascente Milan Italy; ^26^ Department of Cardiology and Angiology University Heart Center Freiburg‐Bad Krozingen Freiburg Germany

**Keywords:** Cardiogenic shock, Heart failure, Non‐AMI CS, Kidney function, Kidney injury

## Abstract

**Aims:**

Heart failure–related cardiogenic shock (HF‐CS) accounts for about half of CS cases, with a paucity of data regarding the role of kidney injury in this subset. This study aims to evaluate patient characteristics and outcome associated with renal function in patients with HF‐CS.

**Methods and results:**

In this multicentre, international, retrospective study, patients with HF‐CS from 16 tertiary care centres in five countries were enrolled between 2010 and 2021. To investigate differences in clinical presentation, complications, and 30‐day mortality, based on renal function, adjusted logistic and Cox regression models were fitted. Among 1010 HF‐CS patients, the median age was 64 (interquartile range [IQR] 52–75) years, with 71.7% being male. Median baseline creatinine was 1.7 (IQR 1.2–2.5) mg/dl, corresponding to an estimated glomerular filtration rate (eGFR) of 41.0 (IQR 25.2–62.2) ml/min/1.73 m^2^. In patients with acute kidney injury (AKI), 30‐day mortality increased with AKI stages (no AKI 41.7%, AKI stage 1 43.3%, AKI stage 2 50.0%, AKI stage 3 63.7%; adjusted hazard ratio [HR] for AKI stage 3 1.97, 95% confidence interval [CI] 1.56–2.48, *p* < 0.001). Similarly, severe renal dysfunction (eGFR ≤ median) was associated with a 21% higher 30‐day mortality risk (61.0% vs. 40.1%; adjusted HR 1.48, 95% CI 1.20–1.84, *p* < 0.001). Sepsis and bleeding were associated with both AKI and renal dysfunction, even after adjustment.

**Conclusions:**

In HF‐CS, kidney injury is associated with higher 30‐day mortality, potentially mediated by bleeding and sepsis. These findings support the consideration of kidney function as a prognostic marker and call for the development and evaluation of kidney‐restoring adjunct interventions in HF‐CS.

## Introduction

Cardiogenic shock (CS) represents a critical condition characterized by a sudden decrease in cardiac output, resulting in life‐threatening end‐organ hypoperfusion.^1^, ^2^ Besides acute myocardial infarction (AMI) as the underlying cause of CS, nearly half of CS cases are due to heart failure (HF), with mortality rates persistently high at 30–50%.^3^, ^4^, ^5^, ^6^, ^7^, ^8^, ^9^, ^10^, ^11^ Due to the broad heterogeneity in the underlying pathology, patients with HF‐CS remain a clinical challenge, particularly concerning clinical evaluation and tailored application of CS‐targeted therapies.^2^, ^12^, ^13^, ^14^ Persisting gaps in clinical trials result in a scarcity of precise treatment recommendations for HF‐CS.^1^

CS can cause acute and chronic kidney injury.^15^, ^16^ The pathophysiology of acute kidney injury (AKI) in this setting emerges from a combination of reduced arterial perfusion, venous congestion, neurohormonal dysregulation, and compromised autoregulation, including ischaemic and inflammatory processes at a microvascular level.^15^, ^17^, ^18^ Studies suggest that the incidence of AKI complicating CS is high, ranging from 20% to 35%, and is independently associated with higher mortality.^15^, ^16^, ^19^, ^20^, ^21^ Most of the published data describe AKI in patients with AMI. In AMI, vascular disease and exposure to contrast agents can contribute to AKI.^22^ In HF‐CS, the prevalence and prognostic impact of AKI is not well known.^18^, ^19^ In this context, it remains inadequately explored whether the use of mechanical circulatory support (MCS) devices improves renal dysfunction.^23^, ^24^

The objective of this study was to quantify the prevalence of AKI in patients with HF‐CS and to determine baseline characteristics, in‐hospital complications, and mortality associated with baseline renal function in this situation.

## Methods

### Data

All analyses were performed in an international, multicentre, observational, anonymized dataset collecting information from patients with HF‐CS treated in 16 international tertiary care centres, specifically targeting patients with HF‐CS. These patients were treated conservatively, with Impella (Impella® device family, Abiomed, Danvers, MA, USA), veno‐arterial extracorporeal membrane oxygenation (VA‐ECMO), or Impella plus VA‐ECMO. Data on patients treated with other MCS devices, such as intra‐aortic balloon pump, were not collected. Detailed information regarding data entry procedures, definitions of CS, and the inclusion/exclusion criteria for this non‐ischaemic CS registry have been published.^12^, ^14^, ^25^

Data from patients with HF‐CS enrolled between 2010 and 2021 were analysed. Eligibility for this study required patients to present with CS as defined by the Society for Cardiovascular Angiography and Interventions (SCAI), according to the initial consensus document published in 2019.^26^ Local investigators extracted the data from patient records and retrospectively assigned the SCAI classification. Patients presenting with AMI, diagnosed by the local treating physicians based on clinical assessment, electrocardiographic findings, and serial troponin measurements, along with transthoracic echocardiography and coronary angiography when indicated, were excluded from this study. Patients with a need for urgent coronary revascularization, irrespective of feasibility, were also excluded. Additional exclusion criteria encompassed CS primarily attributed to right HF (e.g. acute pulmonary embolism), VA‐ECMO‐assisted resuscitation, post‐cardiotomy CS, or conditions associated with a life expectancy of less than 6 months.

This analysis adheres to the principles outlined in the Declaration of Helsinki and received approval from local ethics committees. The main ethics committee waived the need for informed consent due to the study retrospective nature and its reliance on completely anonymized data.

### Definition of study groups

Patients with HF‐CS were stratified by four definitions of kidney injury at baseline (online supplementary *Table* *S1*). First, creatinine‐based renal dysfunction (RD*crea*) was determined using a median cut‐off value. Second, creatinine‐based AKI (AKI*crea*) was defined, adapted from the Kidney Disease: Improving Global Outcomes (KDIGO) criteria, by an increase in creatinine from baseline to 24‐h value: a ≥50% increase in creatinine or an increase of ≥0.3 mg/dl for stage 1, a 100–199% increase in creatinine for stage 2, and a ≥200% increase in creatinine or a serum creatinine level >4 mg/dl for stage 3, or the initiation of renal replacement therapy (RRT). Due to diuretic treatment in many patients with HF‐CS, which influences urine output, and often inaccurate urine output determinations in emergency settings, AKI was analysed solely based on creatinine measurements. Third, estimated glomerular filtration rate (eGFR)‐based renal dysfunction (RD*gfr*) at baseline was assessed using a median cut‐off, with eGFR calculated by the 2021 Chronic Kidney Disease Epidemiology Collaboration (CKD‐EPI) equation.^27^, ^28^ Fourth, eGFR‐based AKI (AKI*gfr*) was defined by the decrease in eGFR from baseline to 24‐h value, calculated using the CKD‐EPI equation.

### Outcome

The primary outcome of this study was the cumulative all‐cause 30‐day mortality. In‐hospital complications were assessed as secondary outcomes as follows: bleeding events were stratified into moderate and severe, as defined by the Global Utilization of Streptokinase and Tissue Plasminogen Activator for Occluded Coronary Arteries (GUSTO) criteria; intracerebral bleeding, haemorrhagic stroke, ischaemic stroke, and hypoxic brain damage were identified using computed tomography; intervention due to bleeding; intervention due to access site‐related ischaemia; laparotomy due to abdominal compartment or bowel ischaemia; haemolysis, defined as lactate dehydrogenase ≥1000 U/L and haptoglobin <0.3 g/L in two samples within 24 h; RRT; sepsis, defined by systemic inflammatory response syndrome criteria and ≥2 positive blood cultures; pulmonary oedema identified by radiographic imaging.

### Statistical analyses

Binary variables are presented as absolute numbers and relative frequencies, and comparisons were conducted using Fisher's exact test. Continuous variables are shown as the median with interquartile range (IQR) and analysed using the Kruskal–Wallis test.

To evaluate the association of clinical characteristics during the index event, comorbidities and in‐hospital complications, in patients with severe renal dysfunction on admission (RD*crea*, RD*gfr*) versus higher stages of AKI (AKI*crea*, AKI*gfr*) versus those with better renal function on admission or lower stages of AKI, multivariable mixed‐effects logistic regression models with centre as a random intercept were fitted. These models were adjusted for age, sex, lactate, pH, and prior cardiopulmonary resuscitation (CPR).

The crude 30‐day mortality rates and survival curves were calculated using the Kaplan–Meier method, with the number of individuals at risk reported. Comparisons between groups were conducted using the log‐rank test. To evaluate the association between RD*crea*, RD*gfr*, AKI*crea*, AKI*gfr* and the use of RRT with mortality risk, cohort‐stratified Cox proportional hazard regression models were fitted. These models were adjusted for age, sex, lactate, pH, and prior CPR. To evaluate the impact of renal function on mortality in patients with HF‐CS undergoing MCS, an interaction term was added, stratified by the median RD*gfr* cut‐off. In addition, subgroup analyses were performed for specific MCS modalities, including Impella only, Impella combined with VA‐ECMO, and VA‐ECMO only. To assess the impact of HF‐CS modalities (de novo vs. acute‐on‐chronic) on mortality in patients with severe renal dysfunction, an interaction term was used to compare mortality risk between groups.

To evaluate the dynamics of renal function, trajectories of serum creatinine values in subgroups over a 7‐day period from baseline were illustrated and comparatively analysed using the Kruskal–Wallis test.

Hazard ratios (HR), odds ratios (OR) and 95% confidence intervals (CI) are presented, and a *p*‐value of <0.05 was considered statistically significant. Analyses were performed using R statistical software (version 4.3.1).

## Results

### Study cohort

A total of 1010 patients with HF‐CS were eligible for the final analysis. The median age of the cohort was 64 years (IQR 52–75), and 71.7% were male. Of these patients, 477 (47.2%) presented with severe de novo HF, and 533 (52.8%) with acute‐on‐chronic HF. A total of 569 (57.5%) patients had a history of arterial hypertension, 264 (26.5%) of diabetes mellitus, and 434 (44.0%) of atrial fibrillation. Ischaemic cardiomyopathy was present in 243 patients (34.0%).

At the index event, the median baseline creatinine level was 1.7 mg/dl (IQR 1.2–2.5), corresponding to an eGFR of 41.0 ml/min/1.73 m^2^ (IQR 25.2–62.2). Baseline arterial lactate was 5.0 mmol/L (IQR 2.7–8.6), the pH value was 7.30 (IQR 7.20–7.40), and the left ventricular ejection fraction (LVEF) was 20% (IQR 15–30). A total of 386 (38.4%) patients had a prior cardiac arrest, 398 (39.4%) were treated with MCS, 653 (66.0%) patients were on mechanical ventilation, with a median Horowitz index (PaO_2_/FiO_2_) of 190 (IQR 103–290). Baseline characteristics for the overall cohort and stratified by median baseline eGFR are detailed in *Table* *1* and stratified by median baseline creatinine in online supplementary *Table* *S2*.

**Table 1:** Characteristics for the overall cohort and stratified by renal function at baseline (estimated glomerular filtration rate >41.0 vs. ≤41.0 ml/min/1.73 m^2^)

	All (*n* = 1010)	Missing data (%)	eGFR >41.0 ml/min/1.73 m^2^ (*n* = 505)	eGFR ≤41.0 ml/min/1.73 m^2^ (*n* = 505)	*p*‐value
Demographics					
Age, years	64.0 (52.0–75.0)	0	61.0 (46.0–73.0)	67.0 (57.0–76.0)	<0.001
Male sex, *n* (%)	724 (71.7)	0	354 (70.1)	370 (73.3)	0.29
Medical history					
Atrial fibrillation	434 (44.0)	2.4	179 (36.5)	255 (51.4)	<0.001
Diabetes mellitus	264 (26.5)	1.5	104 (20.8)	160 (32.3)	<0.001
Arterial hypertension	569 (57.5)	1.2	257 (52.1)	312 (62.8)	0.001
Body mass index, kg/m^2^	26.2 (23.4–30.1)	4.1	25.2 (22.7–29.0)	27.3 (24.1–31.2)	<0.001
History of known heart failure	533 (52.8)	0	214 (42.4)	319 (63.2)	<0.001
HFrEF	473 (83.7)	44.1	200 (84.7)	273 (83.0)	0.64
HFpEF	31 (5.5)	44.1	10 (4.2)	21 (6.4)	0.35
Ischaemic cardiomyopathy	243 (34.0)	29.2	94 (29.3)	149 (37.8)	0.017
Prior coronary revascularization	244 (25.3)	4.7	100 (20.4)	144 (30.4)	<0.001
Clinical presentation					
Systolic blood pressure, mmHg (worst value within 6 h)	82.0 (70.0–92.0)	1.7	83.0 (71.0–95.0)	80.0 (70.0–90.0)	0.15
Diastolic blood pressure, mmHg (worst value within 6 h)	50.0 (40.0–57.0)	2.2	50.0 (40.0–60.0)	50.0 (40.0–55.5)	0.10
Mean arterial blood pressure, mmHg	60.5 (53.0–70.0)	38.4	63.0 (54.1–70.5)	60.0 (51.0–68.0)	0.002
Vasopressor use	878 (87.0)	0.1	425 (84.2)	453 (89.9)	0.009
Heart rate, bpm (worst value within 6 h)	96.0 (76.0–120.0)	1.5	96.0 (77.0–120.0)	96.0 (76.0–120.0)	0.23
Lactate, mmol/L (worst value within 6 h)	5.0 (2.7–8.6)	8.1	4.0 (2.5–7.3)	6.2 (3.0–9.7)	<0.001
pH (worst value within 6 h)	7.3 (7.2–7.4)	3.9	7.3 (7.2–7.4)	7.3 (7.2–7.4)	0.004
Prior CPR	386 (38.4)	0.6	210 (41.9)	176 (35.0)	0.027
Mechanical ventilation	653 (66.0)	2.0	330 (66.8)	323 (65.1)	0.59
Horowitz index (worst value within 6 h)	190.0 (103.0–290.0)	29.8	187.0 (109.5–293.0)	190.9 (98.0–288.5)	0.57
Creatinine, mg/dl (worst value within 6 h)	1.7 (1.2–2.5)	0	1.2 (1.0–1.4)	2.5 (2.1–3.5)	<0.001
SCAI CS class		3.1			
B	147 (15.0)		92 (18.9)	55 (11.2)	0.001
C	333 (34.0)		172 (35.3)	161 (32.7)	0.42
D	237 (24.2)		119 (24.4)	118 (24.0)	0.88
E	262 (26.8)		104 (21.4)	158 (32.1)	<0.001
Mechanical circulatory support					
Mechanical circulatory support, *n* (%)	398 (39.4)	0	200 (39.6)	198 (39.2)	0.95
Only VA‐ECMO, *n* (%)	168 (16.6)	0	80 (15.8)	88 (17.4)	0.55
Impella + VA‐ECMO, *n* (%)	89 (8.8)	0	40 (7.9)	49 (9.7)	0.37
Only Impella, *n* (%)	141 (14.0)	0	80 (15.8)	61 (12.1)	0.10

Continuous variables are shown as a median (25th–75th percentile), with the *p*‐value calculated using the Kruskal–Wallis test. Binary variables are shown as absolute and relative frequencies, with the *p*‐value calculated by Fisher's exact test.

CPR, cardiopulmonary resuscitation; CS, cardiogenic shock; eGFR, estimated glomerular filtration rate (by the 2021 Chronic Kidney Disease Epidemiology Collaboration equation); HFrEF, heart failure with reduced ejection fraction; HFpEF, heart failure with preserved ejection fraction; SCAI, Society for Cardiovascular Angiography and Interventions; VA‐ECMO, veno‐arterial extracorporeal membrane oxygenation.

### Clinical presentation characteristics associated with renal dysfunction and acute kidney injury

**Figure 1:**
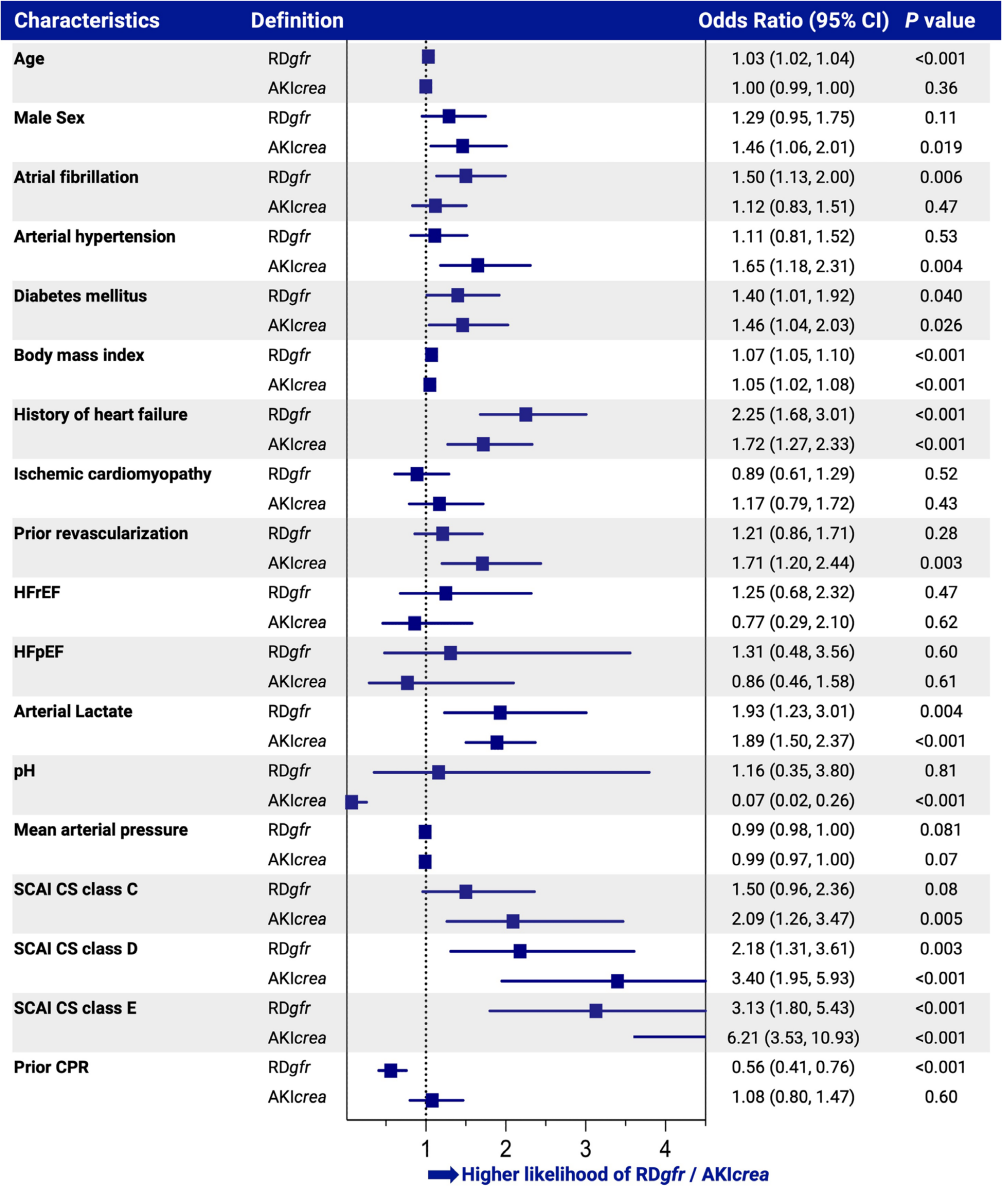
Associations between demographics, medical history, clinical presentation, and renal dysfunction or acute kidney injury. Odds ratio calculated by mixed effects logistic regressions, adjusted for age, sex, lactate, pH, and prior cardiopulmonary resuscitation (CPR). Definition details of renal dysfunction (RD*gfr*) and acute kidney injury (AKI*crea*) are provided in online supplementary *Table* *S1*. CI, confidence interval; CS, cardiogenic shock; HFpEF, heart failure with preserved ejection fraction; HFrEF, heart failure with reduced ejection fraction; SCAI, Society for Cardiovascular Angiography and Interventions.

In patients with RD*gfr* or AKI*crea* a higher body mass index (adjusted OR 1.07, 95% CI 1.05–1.10, *p* < 0.001 for RD*gfr*; adjusted OR 1.05, 95% CI 1.02–1.08, *p* < 0.001 for AKI*crea*) and a history of HF (adjusted OR 2.25, 95% CI 1.68–3.01, *p* < 0.001 for RD*gfr*; adjusted OR 1.72, 95% CI 1.27–2.33, *p* < 0.001 for AKI*crea*) were more likely. Additionally, both RD*gfr* and AKI*crea* were associated with higher lactate levels (adjusted OR 1.93, 95% CI 1.23–3.01, *p* < 0.001 for RD*gfr*; adjusted OR 1.89, 95% CI 1.50–2.37, *p* < 0.001 for AKI*crea*) and advanced shock severity, SCAI CS stage D (adjusted OR 2.18, 95% CI 1.31–3.61, *p* = 0.003 for RD*gfr*; adjusted OR 3.4, 95% CI 1.95–5.93, *p* < 0.001 for AKI*crea*) and stage E (adjusted OR 3.13, 95% CI 1.80–5.43, *p* < 0.001 for RD*gfr*; adjusted OR 6.21, 95% CI 3.53–10.93, *p* < 0.001 for AKI*crea*). An overview of the clinical presentation characteristics associated with RD*gfr* and AKI*crea* is illustrated in *Figure* *1* (association with RD*crea* in online supplementary *Table* *S3*).

**Figure 2:**
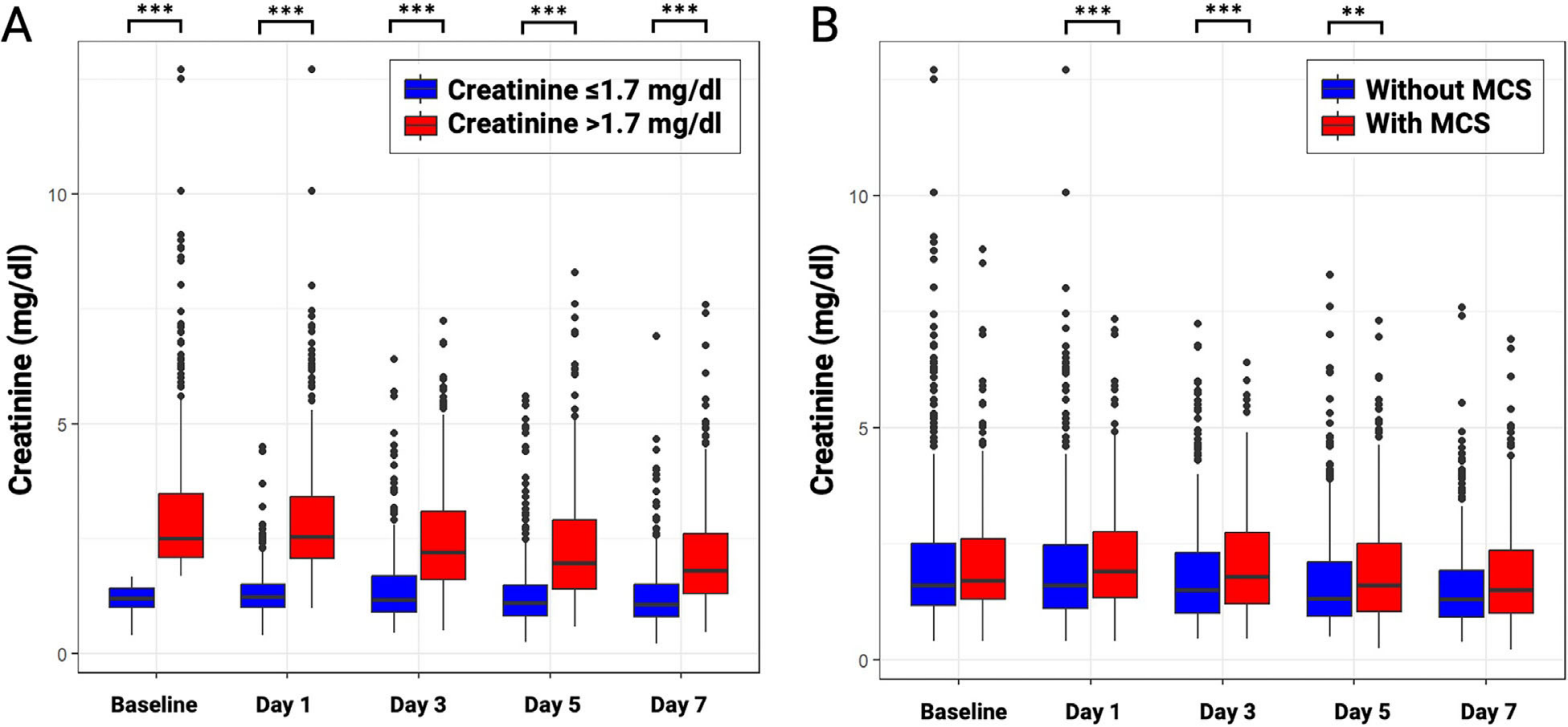
Creatinine trajectories from baseline to day 7, stratified by median baseline creatinine (*A*), and with versus without mechanical circulatory support (MCS) use (*B*). **p < 0.01; ***p < 0.001.

Creatinine trajectories from baseline to day 7 in patients stratified by baseline RD*crea* were investigated and are illustrated in *Figure* *2A*. Patients with lower baseline creatinine (median baseline creatinine 1.2 mg/dl) consistently maintained low creatinine levels over the first 7 days post‐index event. In contrast, patients with higher baseline creatinine levels (median baseline creatinine 2.5 mg/dl) exhibited persistently elevated creatinine levels after 24 h, followed by a gradual decline until day 7, with values remaining significantly higher over time (creatinine lower vs. higher RD*crea*: baseline 1.2 vs. 2.5 mg/dl, *p* < 0.001; day 1 1.2 vs. 2.5 mg/dl, *p* < 0.001; day 3 1.2 vs. 2.2 mg/dl, *p* < 0.001; day 5 1.1 vs. 2.0 mg/dl, *p* < 0.001; day 7 1.1 vs. 1.8 mg/dl, *p* < 0.001).

### Mortality associated with RD*crea* , RD*gfr* , AKI*crea* , AKI*gfr* and renal replacement therapy

**Figure 3:**
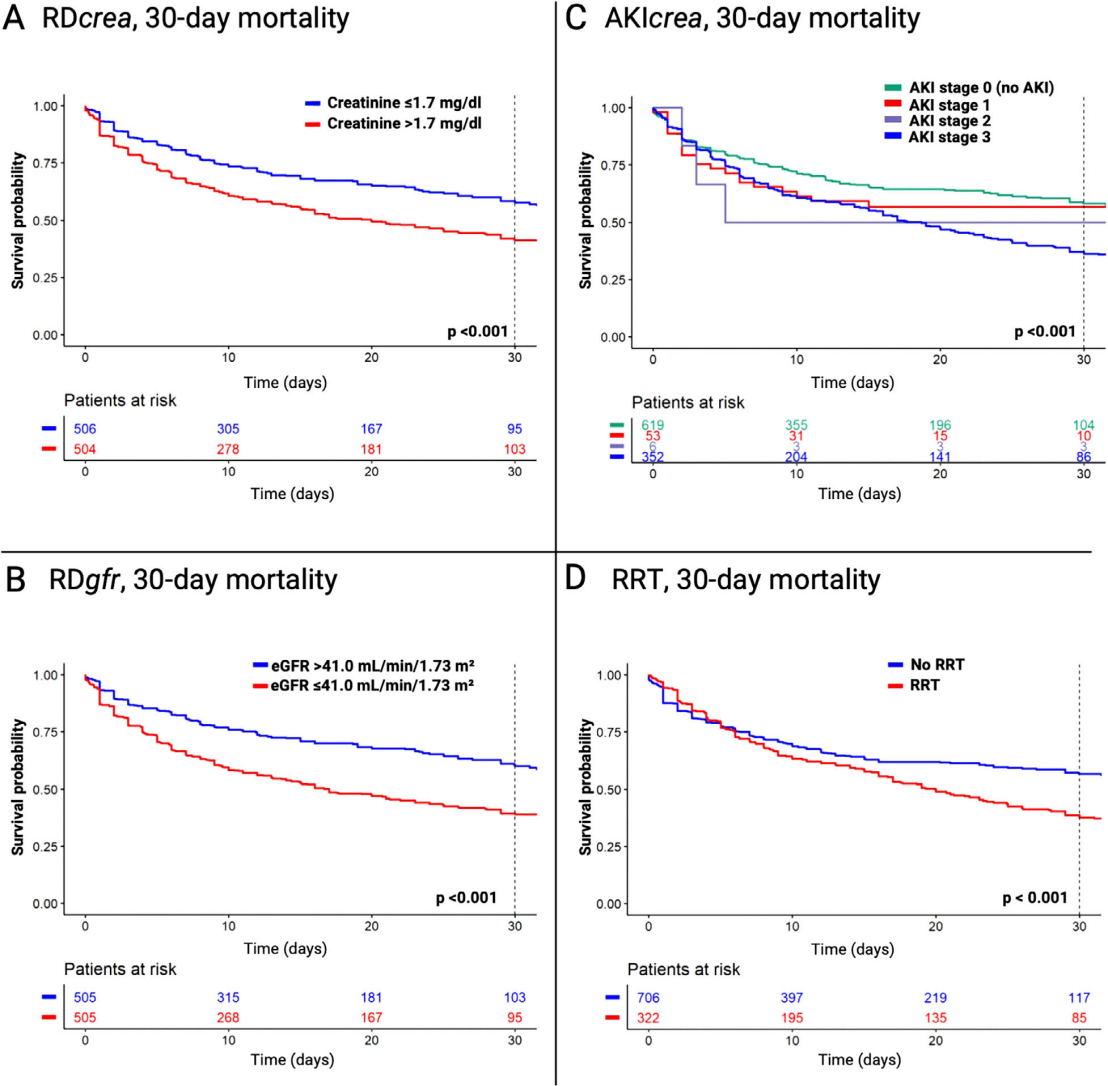
Kaplan–Meier estimates for 30‐day all‐cause mortality in patients with versus without renal dysfunction (RD), acute kidney injury (AKI), or renal replacement therapy (RRT). (*A*) RD*crea*, comparison of patients based on median baseline creatinine levels. (*B*) RD*gfr*, comparison of patients based on median baseline estimated glomerular filtration rate (eGFR). (*C*) AKI*crea*, comparison of patients with AKI stage 0 versus 1 versus 2 versus 3 (definitions of AKI stages are provided in online supplementary *Table* *S1*). (*D*) Comparison of patients with versus no use of RRT.

In the overall study cohort, 394 (43.0%) patients died during a median follow‐up of 24 (IQR 23–27) days. The crude 30‐day mortality was 51.1%. In patients with RD*crea*, the crude 30‐day mortality rate was 42.2% for those with a serum creatinine ≤1.7 mg/dl, compared to 58.8% for those with a serum creatinine >1.7 mg/dl, resulting in an absolute mortality difference of 16.6% (*Figure* *3A*). After adjusting for relevant confounders, increased RD*crea* was associated with a 37% higher relative risk for 30‐day mortality (adjusted HR 1.37, 95% CI 1.11–1.70, *p* = 0.004) (*Table* *2*). Similarly, stratified according to RD*gfr*, the crude 30‐day mortality rate was 61.0% for patients with an eGFR ≤41.0 ml/min/1.73 m^2^, compared to 40.1% for those with an eGFR >41.0 ml/min/1.73 m^2^, resulting in an absolute mortality difference of 21% (*Figure* *3B*). RD*gfr* was associated with a 48% higher relative risk for 30‐day mortality (adjusted HR 1.48, 95% CI 1.20–1.84, *p* < 0.001) (*Table* *2*). While RD*gfr* was associated with mortality, there was no difference in its impact between patients with de novo HF‐CS and those with acute‐on‐chronic HF‐CS in the adjusted interaction term analysis (online supplementary *Table* *S4*).

**Table 2:** Unadjusted and adjusted associations of renal dysfunction, acute kidney injury, and renal replacement therapy with 30‐day mortality

Definition	HR (95% CI)	*p*‐value
RD*crea*		
Unadjusted	1.64 (1.35–1.98)	<0.001
Model 1	1.56 (1.28–1.90)	<0.001
Model 2	1.37 (1.11–1.70)	0.004
RD*gfr*		
Unadjusted	1.91 (1.57–2.32)	<0.001
Model 1	1.63 (1.34–1.99)	<0.001
Model 2	1.48 (1.20–1.84)	<0.001
AKI*crea* stage 1		
Unadjusted	1.14 (0.72–1.81)	0.56
Model 1	1.13 (0.71–1.78)	0.61
Model 2	1.29 (0.77–2.15)	0.33
AKI*crea* stage 2		
Unadjusted	1.06 (0.33–3.41)	0.92
Model 1	0.99 (0.31–3.20)	0.99
Model 2	0.91 (0.22–3.83)	0.90
AKI*crea* stage 3		
Unadjusted	1.70 (1.39–2.08)	<0.001
Model 1	1.79 (1.46–2.20)	<0.001
Model 2	1.97 (1.56–2.48)	<0.001
AKI*gfr*		
Unadjusted	0.91 (0.70–1.18)	0.49
Model 1	0.88 (0.67–1.14)	0.33
Model 2	0.84 (0.63–1.11)	0.22
Use of RRT		
Unadjusted	1.41 (1.16–1.70)	<0.001
Model 1	1.52 (1.25–1.84)	<0.001
Model 2	1.35 (1.10–1.66)	0.005

AKI, acute kidney injury; CI, confidence interval; HR, hazard ratio; RD, renal dysfunction; RRT, renal replacement therapy.

Cohort‐stratified Cox proportional hazard regression models are shown (model 1, adjusted for age and sex; model 2 adjusted for age, sex, lactate, pH, and prior cardiopulmonary resuscitation). A detailed explanation of the definitions for RD and AKI is provided in online supplementary *Table* *S1*.

There was a stepwise increase in the crude 30‐day mortality rate with advancing AKI stages 0–3 (41.7% vs. 43.3% vs. 50.0% vs. 63.7%) (*Figure* *3C*), with an adjusted HR for severe AKI stage 3 vs. 0 of 1.97 (95% CI 1.56–2.48, *p* < 0.001) (*Table* *2*). In patients treated with versus without RRT, 30‐day mortality was 62.3% vs. 43.3% (*Figure* *3D*), with a corresponding adjusted HR of 1.35 (95% CI 1.10–1.66, *p* = 0.005).

The eGFR dynamic within the first 24 h was not associated with 30‐day mortality (*Table* *2*, online supplementary *Figure* *S1*).

### In‐hospital complications associated with renal dysfunction and acute kidney injury

**Figure 4:**
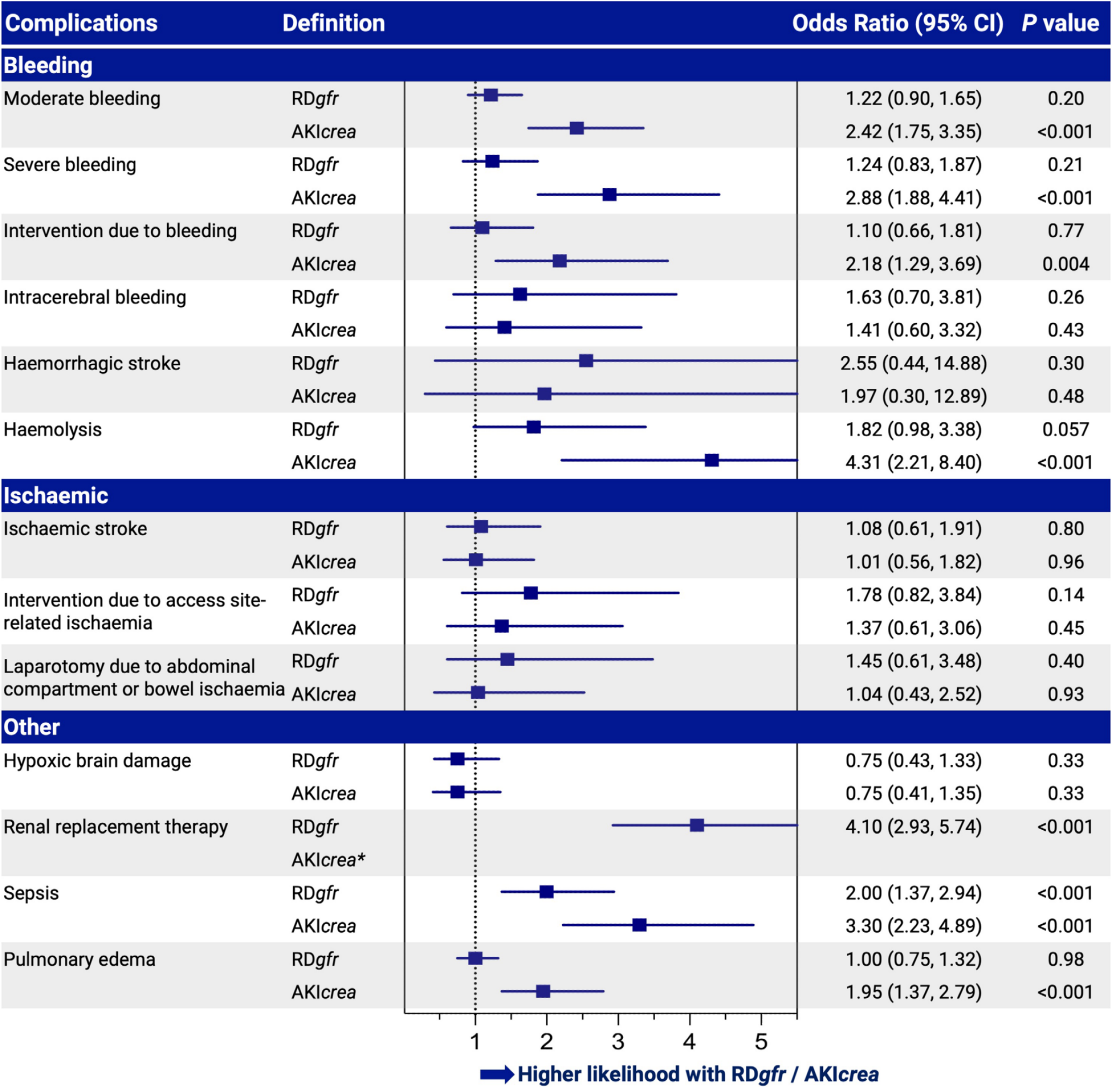
Associations between in‐hospital complications and renal dysfunction or acute kidney injury. Odds ratio calculated by mixed effects logistic regressions, adjusted for age, sex, lactate, pH, and prior cardiopulmonary resuscitation. Definition details of renal dysfunction (RD*gfr*) and acute kidney injury (AKI*crea*) are provided in online supplementary *Table* *S1*. CI, confidence interval. *Renal replacement therapy is included in the acute kidney injury stage 3 definition.

In the overall cohort, stratified by RD*gfr*, complications such as sepsis (21.1% vs. 14.3%, *p* = 0.005) and the need for RRT (44.5% vs. 18.8%, *p* < 0.001) occurred more frequently in patients with severe renal dysfunction (*Table* *3*). After adjustment for relevant confounders, both RD*gfr* and AKI*crea* were associated with an increased risk of sepsis (adjusted OR 2.00, 95% CI 1.37–2.94, *p* < 0.001 for RD*gfr*; adjusted OR 3.30, 95% CI 2.23–4.89, *p* < 0.001 for AKI*crea*), a higher likelihood of requiring RRT (adjusted OR 4.10, 95% CI 2.93–5.74, *p* < 0.001 for RD*gfr*) and a trend towards haemolysis (adjusted OR 1.82, 95% CI 0.98–3.38, *p* = 0.057 for RD*gfr*; adjusted OR 4.31, 95% CI 2.21–8.40, *p* < 0.001 for AKI*crea*) (*Figure* *4*). Additionally, AKI*crea* was significantly associated with moderate (adjusted OR 2.42, 95% CI 1.75–3.35, *p* < 0.001) or life‐threatening bleeding events (adjusted OR 2.88, 95% CI 1.88–4.41, *p* < 0.001), and any surgical intervention due to bleeding (adjusted OR 2.18, 95% CI 1.29–3.69, *p* = 0.004). Complementarily, the association between in‐hospital complications, RD*crea* and AKI*gfr* is detailed in online supplementary *Table* *S5*.

**Table 3:** In‐hospital complications stratified by renal dysfunction at baseline (estimated glomerular filtration rate >41.0 vs. ≤41.0 ml/min/1.73 m^2^)

	All (*n* = 1010)	Missing data (%)	eGFR >41.0 ml/min/1.73 m^2^	eGFR ≤41.0 ml/min/1.73 m^2^	*p*‐value
Bleeding complications					
Moderate bleeding	332 (33.0)	0.5	164 (32.5)	168 (33.6)	0.74
Severe bleeding	147 (14.6)	0.4	70 (13.9)	77 (15.3)	0.53
Intracerebral bleeding	28 (2.9)	3.6	12 (2.4)	16 (3.3)	0.45
Haemorrhagic stroke	7 (0.7)	3.6	3 (0.6)	4 (0.8)	0.72
Intervention due to bleeding	85 (8.4)	0.2	44 (8.7)	41 (8.2)	0.82
Haemolysis	64 (6.4)	0.7	28 (5.6)	36 (7.2)	0.30
Ischaemic complications					
Ischaemic stroke	66 (6.8)	3.7	33 (6.7)	33 (6.9)	1.00
Intervention due to access site‐related ischaemia	35 (3.5)	0.3	16 (3.2)	19 (3.8)	0.61
Laparotomy due to abdominal compartment or bowel ischaemia	23 (2.3)	0.4	10 (2.0)	13 (2.6)	0.54
Other complications					
Hypoxic brain damage	70 (7.2)	4.1	41 (8.4)	29 (6.0)	0.17
Renal replacement therapy	319 (31.6)	0.2	95 (18.8)	224 (44.5)	<0.001
Sepsis	178 (17.7)	0.3	72 (14.3)	106 (21.1)	0.005

Binary variables are shown as absolute and relative frequencies, with the *p*‐value calculated by Fisher's exact test.

Estimated glomerular filtration rate was calculated by the 2021 Chronic Kidney Disease Epidemiology Collaboration equation.

### Impact of selected treatments

Patients with severe RD*gfr* were more frequently treated with vasopressors (89.9% vs. 84.2%). After adjusting for relevant confounders, both patients with RD*gfr* and AKI*crea* were more likely associated with vasopressor treatment (online supplementary *Table* *S6*).

The distribution of MCS use was not different in patients stratified by RD*gfr* (*Table* *1*). RD*gfr* and AKI*crea* were significantly associated with increased MCS use (detailed for MCS subgroups in online supplementary *Table* *S6*). Patients with MCS presented with persistently higher creatinine levels throughout the first 7 days following the index event compared to those without MCS (*Figure* *2B*). The use of MCS was associated with a significantly increased risk of 30‐day mortality in patients with RD*gfr* (eGFR ≤ median: adjusted HR 1.67, 95% CI 1.14–2.44, *p* = 0.008; eGFR > median: adjusted HR 1.41, 95% CI 1.05–1.89, *p* = 0.024), without significant differences in the interaction analysis between GFR categories (interaction‐*p* = 0.430).

## Discussion

In this retrospective, multicentre, international study of 1010 patients with HF‐CS, impaired kidney function prompted presentation with higher shock severity and was associated with significantly higher mortality rates, potentially mediated by a higher risk of complications such as bleeding and sepsis (*Graphical Abstract*). MCS use was more frequently associated with severe kidney injury but did not impact overall outcome. These findings suggest that a precise assessment of renal function at the CS index event could be an important prognostic marker in HF‐CS, indicating that targeted interventions to improve kidney function might enhance patient outcome.

### Association between kidney injury and outcome in heart failure‐related cardiogenic shock

Heart failure‐related CS, characterized by its pathophysiological peculiarities, aetiological modalities (e.g. acute‐on‐chronic HF‐CS vs. de novo HF‐CS), variations in clinical presentation, diverse phenotypes, and distinctions in shock severity, remains a significant clinical challenge.^1^, ^12^, ^29^, ^30^ Moreover, there is a critical lack of high‐quality evidence to support standardized diagnostic protocols, effective risk stratification, and targeted therapeutic interventions in the management of HF‐CS.^1^, ^2^, ^12^, ^13^, ^31^, ^32^ In this context, there are limited data on the cardiorenal interaction, as well as on patient characteristics and outcomes related to renal function in HF‐CS, which was the focus of this study.^18^, ^19^

In this study of patients with HF‐CS, AKI occurred at a notably high incidence, affecting approximately 40% patients, as compared to previous studies on renal dysfunction in AMI‐CS, which have typically ranged from 20% to 35%.^15^, ^16^, ^19^, ^20^, ^21^ Baseline renal function or AKI within the first 24 h after the index event were associated with a higher prevalence of cardiovascular risk factors, cardiac comorbidities, and known acute‐on‐chronic HF. Additionally, these patients were more likely to present with higher lactate levels and advanced SCAI shock stages (D and E), even after adjusting for relevant confounders. These observations, specifically demonstrated in HF‐CS, are consistent with previous reports from an all‐comers CS cohort indicating that patients with more severe AKI tend to have more comorbidities, exhibit greater initial shock severity, and experience more extensive multi‐organ failure.^33^

In previous studies, AMI‐CS complicated by AKI was independently associated with worse outcome.^19^, ^20^, ^34^, ^35^, ^36^ In our analysis, we observed a 21% higher absolute 30‐day mortality rate in patients with an eGFR ≤41.0 ml/min/1.73 m^2^ at the HF‐CS index event, along with a stepwise increase in mortality with more advanced AKI. These findings are in line with data of patients hospitalized with acute HF, where renal dysfunction has been associated with higher mortality rates.^37^, ^38^, ^39^ However, direct comparison of these data is challenging for several reasons. First, there is a paucity of comparable data specifically on primary non‐ischaemic HF‐CS cohorts.^20^, ^34^, ^35^ Second, definitions of AKI in CS exhibit considerable heterogeneity across studies, with variations in creatinine thresholds and different application of KDIGO criteria.^19^, ^20^, ^33^, ^34^, ^35^ Third, treatment regimens differ significantly, including variations in the use of vasopressors, MCS, and associated complication rates, further complicating direct comparisons.^19^, ^20^, ^33^, ^34^, ^35^

Renal dysfunction can adversely affect distant organs and is linked to an increased risk of non‐renal complications, though this relationship remains incompletely characterized in the context of CS.^40^ Our findings indicate that AKI was significantly associated with elevated risks of bleeding complications, sepsis, and the need for RRT. Furthermore, the use of RRT was independently associated with a 35% higher relative risk of 30‐day mortality compared to patients not receiving RRT. This aligns with previous data, showing that AKI requiring dialysis is associated with increased short‐term mortality and higher incidence of in‐hospital bleeding.^40^, ^41^ Bleeding events can be further exacerbated by uraemia and may also be influenced by factors associated with renal dysfunction, such as acute hepatic failure, coagulopathy, or infection.^42^

In the context of early risk prediction for patients with HF‐CS, using baseline creatinine or eGFR might offer advantages over AKI assessment according to KDIGO. Given that creatinine levels and the initiation of RRT in KDIGO are evaluated over several hours to days for AKI staging, this method may not provide timely information crucial for early decision‐making during the early phase of CS. In addition, urine output has been investigated as a criterion for AKI and mortality prediction in previous studies.^19^ However, accurately measuring urine output in clinical CS emergency settings is often challenging, and studies have shown that patients with oliguria are inconsistently correlated with AKI.^19^, ^43^ Furthermore, the likelihood that patients with acute‐on‐chronic HF‐CS may more frequently require RRT due to refractory decongestion complicates the application of the AKI definition according to KDIGO in this heterogeneous HF‐CS cohort. In contrast, baseline creatinine or eGFR offers an easy and immediate assessment of kidney function, which could facilitate earlier risk stratification and timely interventions. Our study suggests that baseline creatinine or eGFR is strongly associated with mortality, highlighting its potential as an early prognostic marker before significant changes in serum creatinine or urine output, as defined by AKI KDIGO, become apparent. Additionally, patients with initially elevated creatinine and reduced eGFR levels exhibited persistently high creatinine levels throughout the first 7 days following the index event, thereby allowing early detection of significant renal dysfunction. In contrast, those with lower baseline creatinine levels experienced only minimal increases over time. Therefore, integrating baseline creatinine or eGFR into the initial CS evaluation might enhance mortality prediction and prompt targeted treatment strategies for renal dysfunction in this CS subset. Larger, in‐depth HF‐CS cohorts are needed to validate these findings and to further assess appropriate cut‐off values for clinical application.

Kidney injury in advanced HF reflects a complex interplay of factors, including reduced arterial perfusion with forward failure, venous congestion, neurohormonal dysregulation, and impaired autoregulation, all contributing to ischaemic and inflammatory processes at the microvascular level.^15^, ^17^, ^18^ We previously demonstrated that patients with acute‐on‐chronic HF‐CS exhibited higher lactate and creatinine trajectories compared to those with de novo HF, indicating a greater extent of subclinical end‐organ damage in the presence of pre‐existing HF.^12^ However, the impact of kidney injury on mortality did not significantly differ between patients with de novo and those with acute‐on‐chronic HF‐CS at the index event. The underlying pathophysiological mechanisms driving renal dysfunction in these two CS subgroups may be distinct. In de novo HF‐CS, renal impairment may be driven by acute forward failure and/or backward failure, depending on whether the right or left ventricle is acutely affected. In contrast, in acute‐on‐chronic HF‐CS, backward failure and neurohormonal activation, accompanied by severe venous congestion, might play a more significant role. This is further supported by recent studies indicating that reduced cardiac index may not be the predominant factor for renal dysfunction in advanced HF; rather, venous congestion appears to be a pivotal determinant in the progression of renal impairment in patients with advanced decompensated HF.^44^, ^45^

### Impact of treatment modalities on kidney injury in heart failure‐related cardiogenic shock

Prevention, timely detection, and early intensive treatment of renal dysfunction in HF‐CS could significantly improve survival outcomes in this high‐risk CS subset, although currently available treatments are primarily indirect, for example, address the restoration of adequate kidney perfusion. However, the impact of vasopressors and MCS devices on renal dysfunction, for example, the treatments most frequently used to control blood pressure and hence kidney perfusion, remains inadequately explored.^23^, ^24^ Notably, higher doses of catecholamines are associated with an increased likelihood of persistent AKI, and while newer agents like levosimendan show promise, they have not yet demonstrated a mortality benefit in HF‐CS.^46^ MCS may offer haemodynamic support and adequate tissue perfusion, but evidence for its efficacy in HF‐CS is still lacking.^14^, ^32^ In this study, impaired kidney function was linked to increased MCS utilization during the hospital course. However, severe renal dysfunction did not impact the outcome of patients with MCS. Several factors may explain these findings. First, in patients with advanced CS severity, the initial CS event may have caused such severe subclinical kidney damage before MCS implantation that even increased tissue perfusion may be insufficient to significantly impact kidney reserve and recovery. Second, the systemic inflammatory response associated with MCS therapy could exacerbate renal injury. Third, given that AKI in acute‐on‐chronic HF‐CS could be predominantly driven by venous congestion, MCS—which primarily addresses forward failure—may not confer substantial benefit in this subset, and can sometimes even worsen venous congestion via overloading the right ventricle. Finally, mechanisms such as coagulopathy and fluid overload during MCS therapy may worsen AKI.

### Limitations

This study is based on non‐randomized data, which is a primary limitation as it precludes drawing causal conclusions. Although all hospitals involved are large tertiary care centres with extensive experience in managing CS and utilizing MCS devices, this expertise could contribute to both a higher use of MCS and a greater prevalence of severe CS cases within this cohort.

Regarding specific limitations in assessing renal function and AKI, it is important to note the lack of reliable data on serum creatinine levels prior to the index hospitalization, as well as insufficient data on prior RRT. However, this reflects the typical emergency setting in CS management, where accurate baseline creatinine values and dialysis histories are often initially unavailable. In addition, the eGFR calculated by the 2021 CKD‐EPI equation may predictably lack accuracy in AKI, limiting its reliability in acute settings. Specifically, it may be unsuitable for 24‐h eGFR assessment due to its reliance on stable creatinine levels and steady‐state assumptions. Consequently, as expected, we observed no significant eGFR changes over 24 h, despite notable creatinine fluctuations. However, to our knowledge, no comparable data are currently available for this CS subset and setting.

Moreover, the impact of furosemide treatment, particularly at high doses, on renal function and serum creatinine levels was inadequately captured in this registry. Furthermore, there is a lack of reliable data on levosimendan use, a therapy increasingly employed in advanced HF management. It would also be of significant interest to correlate our findings with invasive haemodynamic data, particularly to characterize the influence of haemodynamic and congestion profiles on renal function in HF‐CS.

## Conclusion

In this retrospective, multicentre, international study of 1030 patients with HF‐CS, impaired renal function was strongly associated with higher mortality rates. These patients not only had a greater burden of cardiovascular comorbidities and more advanced shock severity but also experienced a higher incidence of severe in‐hospital complications such as sepsis and bleeding events. These findings highlight the importance of precise renal function assessment at the time of the CS index event as a potential prognostic marker in HF‐CS. Targeted interventions aimed at improving kidney function could help reduce complication rates and enhance patient outcome.

### Funding

J.S. is supported by the German Research Foundation (grant number 546376900). P.K. was partially supported by European Union AFFECT‐AF (grant agreement 847770), and MAESTRIA (grant agreement 965286), British Heart Foundation (PG/20/22/35093; AA/18/2/34218), German Center for Cardiovascular Research supported by the German Ministry of Education and Research (DZHK, grant numbers DZHK FKZ 81X2800182, 81Z0710116, and 81Z0710110), German Research Foundation (Ki 509167694), and Leducq Foundation.

**Conflict of interest**: J.S. received travel fees from Abiomed, outside the submitted work. L.F.B. received speaker fees from Abiomed, outside the submitted work. S.B. reports fundings from Abbott Diagnostics, Amarin, AMGEN, AstraZeneca, Bayer, Siemens, and Novartis as well as honoraria for lectures and/or chairs from Abbott, Abbott Diagnostics, Bayer, Bristol Meyers Squibb, Boehringer Ingelheim, Daiichi Sankyo, GSK, LumiraDx, Novartis, Roche Diagnostics, and Thermo Fisher; he is a member of advisory boards and consultant of Thermo Fisher, and he is scientific advisor of Cardio‐CARE, a 100% non‐profit daughter of the Kühne Foundation. J.D. received speaker fees from AstraZeneca, Bayer, Boehringer Ingelheim, Novartis and travel grants from AstraZeneca, Bayer, Daiichi Sankyo. P.K. received research support for basic, translational, and clinical research projects from European Union, British Heart Foundation, Leducq Foundation, Medical Research Council (UK), and German Center for Cardiovascular Research, from several drug and device companies active in atrial fibrillation and has received honoraria from several such companies in the past, but not in the last 5 years; he is listed as inventor on two issued patents held by University of Hamburg (Atrial Fibrillation Therapy WO 2015140571, Markers for Atrial Fibrillation WO 2016012783). S.K. received research support from Cytosorbents and Daiichi Sankyo; lecture fees from ADVITOS, Biotest, CSL Behring, Daiichi Sankyo, Fresenius Medical Care, Gilead, Mitsubishi Tanabe Pharma, MSD, Pfizer, Shionogi and Zoll; and consultant fees from ADVITOS, Fresenius, Gilead, MSD and Pfizer. N.Ma. received personal fees from Edwards Lifesciences, Medtronic, Biotronik, Novartis, Sanofi Genzyme, AstraZeneca, Pfizer, Bayer, Abbott, Abiomed, and Boston Scientific, outside the submitted work. S.M.W. reports Abiomed unrestricted grant for JenaMacs trial; speaker honoraria from Abiomed, Boston Scientific, Pfizer, Daichi Sankyo, outside the submitted work. M.O. reports speaker honoraria and travel compensations from companies Abbott Medical, AstraZeneca, Abiomed, Bayer vital, Biotronik, Bristol Myers Squibb, CytoSorbents, Daiichi Sankyo Deutschland, Edwards Lifesciences Services, Sedana Medical. A.P. reports institutional fees from Getinge and Abiomed, outside the submitted work, and research funding from the Medical Research Council and Barts Charity. T.R. has received honoraria, lecture fees, and grant support from Edwards Lifesciences, AstraZeneca, Bayer, Novartis, Berlin Chemie, Daiichi Sankyo, Boehringer Ingelheim, Novo Nordisk, Cardiac Dimensions, and Pfizer, outside the submitted work; he is co‐founder of Bimyo GmbH, a company that develops cardioprotective peptides, co‐founder of Sygnal GmbH, a company focusing on AI‐based ECG‐algorithms, and co‐founder of Yes2NO, developing nitric oxide‐based treatments. H.R. reports speaker honoraria from Edwards, Abiomed and Medtronic. C.S. reports speaker honoraria from AstraZeneca, outside the submitted work. P.C.S. reports grants from Boehringer Ingelheim, Abiomed Inc, Edwards Inc, Cytosorb Inc, Boston Scientific, and consulting fees and/or honoraria from Bayer, AstraZeneca, Daiichi Sankyo, Novartis, Actelion, Roche, Sanofi Aventis, Pharmacosmos, Medtronic, Thoratec, Boehringer Ingelheim, Heartware, Coronus, Abbott,Boston Scientific, St. Jude Medical, Abiomed and DGK, and trial committee work for Abbott, Abiomed. R.H.G.S reports speaker fees from AstraZeneca, Daiichi Sankyo, Edwards, Bristol Myers Squibb, Pfizer, Bayer Vital, Boehringer Ingelheim. C.Sk. received speaker fees from Abiomed, Boston Scientific and Bristol Myers Squibb. E.B.W. reports grants from Boehringer Ingelheim, and personal fees from Amgen, AstraZeneca, Bayer, Bristol Myers Squibb, Boehringer Ingelheim, CVRx, Daiichi Sankyo, Pfizer, and Novartis, outside the submitted work. D.W. reports speaker fees from Abiomed, AstraZeneca, Bayer, Berlin‐Chemie, Boehringer Ingelheim, Novartis and Medtronic, outside the submitted work. B.S. reports speaker fees from Abiomed, Abbott, AstraZeneca and Inari; and research funding from the DFG, the EKFS, the DZHK and Abiomed, outside the submitted work. All other authors have nothing to disclose.

## Supporting information


**Table S1.** Definitions of study groups based on creatinine and GFR criteria for renal dysfunction and acute kidney injury.
**Table S2.** Baseline characteristics stratified by median baseline creatinine.
**Table S3.** Association between clinical presentation characteristics and RDcrea.
**Table S4.** Impact of de novo versus acute‐on‐chronic HF‐CS in patients with renal dysfunction.
**Table S5.** Association between in‐hospital complications, RDcrea and AKIgfr.
**Table S6.** Association between renal dysfunction, acute kidney injury, and selected treatments modalities.
**Figure S1.** Kaplan–Meier estimates for 30‐day all‐cause mortality in patients with heart failure‐related cardiogenic shock, with verses without eGFR decrease within 24 h.
