# Supplementary material for: Kidney injury in patients with heart failure‐related cardiogenic shock: Results from an international, multicentre cohort study
Source: Eur J Heart Fail. 2025 May 28;27(11):2397–409. doi: 10.1002/ejhf.3701 (PMC12765233; doi:10.1002/ejhf.3701)
Supplement: Supplementary file 1 — Table S1. Definitions of study groups based on creatinine and GFR criteria for renal dysfunction and acute kidney injury. Table S2. Baseline characteristics stratified by median baseline creatinine. Table S3. Association between clinical presentation characteristics and RDcrea. Table S4. Impact of de novo versus acute‐on‐chronic HF‐CS in patients with renal dysfunction. Table S5. Association between in‐hospital complications, RDcrea and AKIgfr. Table S6. Association between renal dysfunction, acute kidney injury, and selected treatments modalities. Figure S1. Kaplan–Meier estimates for 30‐day all‐cause mortality in patients with heart failure‐related cardiogenic shock, with verses without eGFR decrease within 24 h. [file EJHF-27-2397-s001.docx]

***SUPPLEMENTARY APPENDIX***

***Original Research Manuscript:***

**Kidney Injury in Patients with Heart Failure-related Cardiogenic Shock: Results from an International, Multicenter Cohort Study**

Jonas Sundermeyer, MD; Caroline Kellner, MSc; Benedikt N. Beer, MD; Lisa Besch, MD; Angela Dettling, MD; Letizia Fausta Bertoldi, MD; Stefan Blankenberg, MD; Jeroen Dauw, MD; Dennis Eckner, MD; Ingo Eitel, MD; Tobias Graf, MD; Patrick Horn, MD; Joanna Jozwiak-Nozdrzykowska, MD¹; Paulus Kirchhof, MD; Stefan Kluge, MD; Axel Linke, MD; Ulf Landmesser, MD; Enzo Lüsebrink, MD; Nicolas Majunke, MD; Norman Mangner, MD; Sven Möbius Winkler, MD; Peter Nordbeck, MD; Martin Orban, MD; Federico Pappalardo, MD; Matthias Pauschinger, MD; Michal Pazdernik, MD; Alastair Proudfoot, MD, PhD; Matthew Kelham, MD; Tienush Rassaf, MD; Hermann Reichenspurner, MD, PhD; Clemens Scherer, MD; Paul Christian Schulze, MD; Robert H.G. Schwinger, MD; Carsten Skurk, MD; Marek Sramko, MD; Guido Tavazzi, MD; Holger Thiele, MD; Luca Villanova, MD; Nuccia Morici, MD, PhD; Ephraim B. Winzer, MD; Dirk Westermann, MD; and Benedikt Schrage, MD, PhD

***Supplementary Tables:***

- ***Supplementary Table 1:*** *Definitions of study groups based on creatinine and GFR criteria for renal dysfunction and acute kidney injury.*
- ***Supplementary Table 2:*** *Baseline characteristics stratified by median baseline creatinine.*
- ***Supplementary Table 3:*** *Association between clinical presentation characteristics and RDcrea*
- ***Supplementary Table 4:*** *Impact of de novo versus acute-on-chronic HF-CS in patients with renal dysfunction.*
- ***Supplementary Table 5:*** *Association between in-hospital complications, RDcrea and AKIgfr.*
- ***Supplementary Table 6:*** *Association between renal dysfunction, acute kidney injury, and selected treatments modalities.*

***Supplementary Figures:***

- ***Supplementary Figure 1:*** *Kaplan-Meier estimates for 30-day all-cause mortality in patients with heart failure-related cardiogenic shock, with verses without eGFR decrease within 24 h.*

***Supplementary Tables***

***Supplementary Table 1:*** Definitions of study groups based on creatinine and GFR criteria for renal dysfunction and acute kidney injury.

| **Creatinine-based renal dysfunction (RD*crea*)** | Median baseline cut-off value (≤ vs. >) |
| --- | --- |
| **Creatinine-based acute kidney injury (AKI*crea*)** | Adapted from modified Kidney Disease: Improving Global Outcomes (KDIGO) criteria:   \| **Stage 0** – not meeting any criteria of stage 1, 2, 3 \| \| --- \| \| **Stage 1** – any of the following criteria   - Increase in serum creatinine by ≥50-99% within 24 h - Increase in serum creatinine by ≥0.3 mg/dL within 24 h \| \| **Stage 2** – any of the following criteria   - Increase in serum creatinine by 100-199% within 24 h \| \| **Stage 3** – any of the following criteria   - Increase in serum creatinine by ≥200% within 24 h - Serum creatinine ≥4 mg/dL - Renal replacement therapy \| |
| **Glomerular filtration rate (GFR)-based renal dysfunction (RD*gfr*)** | - GFR calculated by the 2021 Chronic Kidney Disease Epidemiology Collaboration (CKD-EPI) formula - Median baseline cut-off value (> vs. ≤) |
| **GFR-based acute kidney injury (AKI*gfr*)** | Decrease in GFR, stratified by decline within 24 h:   - Anny decrease in GFR - ≥10 ml/min/1.73m² - ≥20 ml/min/1.73m² - ≥50 ml/min/1.73m² |

***Supplementary Table 2:*** *Baseline characteristics stratified by median baseline creatinine.*

|  | **All (N=1010)** | **Missing data (%)** | **Creatinine ≤1.7 mg/dl (N=506)** | **Creatinine >1.7 mg/dl (N=504)** | **P value** | |
| --- | --- | --- | --- | --- | --- | --- |
| **Demographics** | | | | | | |
| Age, years | 64.0 (52.0, 75.0) | 0 | 63.0 (49.2, 75.0) | 66.0 (55.0, 74.2) |  | 0.07 |
| Male sex | 724 (71.7) | 0 | 316 (62.5) | 408 (81.0) |  | <0.001 |
| **Medical history** | | | | | | |
| Atrial fibrillation | 434 (44.0) | 2.4 | 174 (35.7) | 260 (52.2) | <0.001 | |
| Diabetes mellitus | 264 (26.5) | 1.5 | 102 (20.5) | 162 (32.6) | <0.001 | |
| Arterial hypertension | 569 (57.5) | 1.9 | 267 (54.2) | 302 (60.8) | 0.040 | |
| Body mass index, kg/m² | 26.2 (23.4, 30.1) | 4.0 | 25.4 (22.9, 29.3) | 27.2 (24.0, 31.0) | <0.001 | |
| History of known heart failure | 533 (52.8) | 0 | 199 (39.3) | 334 (66.3) | <0.001 | |
| HFrEF | 473 (83.7) | 43.2 | 183 (83.6) | 290 (83.8) | 1.00 | |
| HFpEF | 31 (5.5) | 43.2 | 10 (4.6) | 21 (6.1) | 0.57 | |
| Ischemic cardiomyopathy | 243 (34.0) | 28.6 | 90 (28.9) | 153 (37.9) | 0.014 | |
| Prior coronary revascularization | 244 (25.3) | 4.6 | 98 (20.0) | 146 (30.9) | 0.001 | |
| **Clinical presentation** | | | | | | |
| Systolic blood pressure, mmHg (worst value within 6 hours) | 82.0 (70.0, 92.0) | 1.6 | 83.0 (70.8, 95.0) | 80.0 (70.0, 90.0) | 0.11 | |
| Diastolic blood pressure, mmHg (worst value within 6 hours) | 50.0 (40.0, 57.0) | 2.1 | 50.0 (40.0, 58.2) | 50.0 (40.0, 56.0) | 0.45 | |
| Mean arterial blood pressure | 60.5 (53.0, 70.0) | 37.7 | 63.0 (54.0, 70.0) | 60.0 (51.2, 69.0) | 0.010 | |
| Vasopressor use | 878 (87.0) | 0.1 | 429 (84.8) | 449 (89.3) | 0.039 | |
| Heart rate, bpm (worst value within 6 hours) | 96.0 (76.0, 120.0) | 1.5 | 96.0 (76.0, 120.0) | 98.0 (76.0, 120.0) | 0.99 | |
| Lactate, mmol/l (worst value within 6 hours) | 5.0 (2.7, 8.6) | 8.0 | 4.1 (2.5, 7.3) | 6.1 (3.0, 9.8) | <0.001 | |
| pH (worst value within 6 hours) | 7.3 (7.2, 7.4) | 3.8 | 7.3 (7.2, 7.4) | 7.3 (7.2, 7.4) | 0.017 | |
| Prior CPR | 386 (38.4) | 0.6 | 215 (42.8) | 171 (34.1) | 0.005 | |
| Mechanical ventilation | 653 (66.0) | 1.9 | 332 (66.8) | 321 (65.1) | 0.59 | |
| Horowitz index (worst value within 6 hours) | 190.0 (103.0, 290.0) | 29.2 | 188.0 (109.2, 294.0) | 190.8 (96.3, 284.0) | 0.39 | |
| Creatinine, mg/dl (worst value within 6 hours) | 1.7 (1.2, 2.5) | 0 | 1.2 (1.0, 1.4) | 2.5 (2.1, 3.5) | <0.001 | |
| SCAI CS class |  | 3.0 |  |  |  |  |
| B | 147 (15.0) |  | 91 (18.6) | 56 (11.4) | 0.002 | |
| C | 333 (34.0) |  | 177 (36.3) | (31.8) | 0.14 | |
| D | 237 (24.2) |  | 117 (24.0) | 120 (24.4) | 0.88 | |
| E | 262 (26.8) |  | 103 (21.1) | 159 (32.4) | <0.001 | |
| **Mechanical Circulatory Support** | | | | | | |
| Mechanical circulatory support No. (%) | 398 (39.4) | 0 | 191 (37.7) | 207 (41.1) | 0.30 | |
| Only VA-ECMO No. (%) | 168 (16.6) | 0 | 72 (14.2) | 96 (19.0) | 0.43 | |
| Impella + VA-ECMO No. (%) | 89 (8.8) | 0 | 41 (8.1) | 48 (9.5) | 0.44 | |
| Only Impella No. (%) | 141 (14.0) | 0 | 78 (15.4) | 63 (12.5) | 0.20 | |

Continuous variables are shown as a median (25th, 75th percentile), the p-value given is calculated using the Kruskal-Wallis test. Binary variables are shown as absolute and relative frequencies, the P value given is calculated by Fisher`s exact test. CPR, cardiopulmonary resuscitation; HFrEF, heart failure with reduced ejection fraction; HFpEF, heart failure with preserved ejection fraction; SCAI CS class, Society for Cardiovascular Angiography & Interventions Cardiogenic chock class; VA-ECMO, veno-arterial extracorporeal membrane oxygenation.

***Supplementary Table 3:*** *Association between clinical presentation characteristics and RDcrea*.

| Characteristics |  | OR (95% CI) | p-value |
| --- | --- | --- | --- |
| Age | Unadjusted | 1.01 (1, 1.02) | 0.002 |
|  | Adjusted | 1.01 (1.01, 1.02) | 0.001 |
| Male sex | Unadjusted | 2.6 (1.94, 3.47) | <0.001 |
|  | Adjusted | 2.78 (2.03, 3.81) | <0.001 |
| Atrial fibrillation | Unadjusted | 2.02 (1.55, 2.62) | <0.001 |
|  | Adjusted | 1.73 (1.3, 2.31) | <0.001 |
| Arterial hypertension | Unadjusted | 1.36 (1.05, 1.78) | 0.021 |
|  | Adjusted | 1.12 (0.82, 1.52) | 0.49 |
| Diabetes mellitus | Unadjusted | 1.89 (1.42, 2.53) | <0.001 |
|  | Adjusted | 1.65 (1.19, 2.28) | 0.002 |
| Body mass index | Unadjusted | 1.06 (1.04, 1.09) | <0.001 |
|  | Adjusted | 1.06 (1.03, 1.09) | <0.001 |
| Known history of heart failure | Unadjusted | 3.03 (2.34, 3.92) | <0.001 |
|  | Adjusted | 2.67 (1.97, 3.61) | <0.001 |
| Ischemic cardiomyopathy | Unadjusted | 1.5 (1.09, 2.08) | 0.014 |
|  | Adjusted | 1.07 (0.73, 1.56) | 0.73 |
| Prior revascularisation | Unadjusted | 1.87 (1.38, 2.54) | <0.001 |
|  | Adjusted | 1.41 (1, 1.99) | 0.051 |
| HFrEF | Unadjusted | 1.24 (0.71, 2.16) | 0.45 |
|  | Adjusted | 1.18 (0.64, 2.19) | 0.59 |
| HFpEF | Unadjusted | 1.66 (0.66, 4.17) | 0.28 |
|  | Adjusted | 1.42 (0.52, 3.88) | 0.49 |
| Lactate | Unadjusted | 1.68 (1.41, 2.01) | <0.001 |
|  | Adjusted | 1.5 (0.96, 2.35) | 0.074 |
| pH | Unadjusted | 0.35 (0.15, 0.82) | 0.015 |
|  | Adjusted | 1.3 (0.4, 4.26) | 0.67 |
| Mean arterial pressure | Unadjusted | 0.99 (0.98, 1) | 0.034 |
|  | Adjusted | 0.99 (0.98, 1) | 0.20 |
| SCAI CS class C | Unadjusted | 1.38 (0.91, 2.08) | 0.13 |
|  | Adjusted | 1.35 (0.86, 2.11) | 0.20 |
| SCAI CS class D | Unadjusted | 1.79 (1.15, 2.77) | 0.010 |
|  | Adjusted | 2.04 (1.23, 3.39) | 0.006 |
| SCAI CS class E | Unadjusted | 3.03 (1.95, 4.71) | <0.001 |
|  | Adjusted | 2.77 (1.6, 4.81) | <0.001 |
| Prior CPR | Unadjusted | 0.69 (0.54, 0.9) | 0.006 |
|  | Adjusted | 0.53 (0.39, 0.73) | <0.001 |

Odds ratio calculated by mixed effects logistic regressions, adjusted by age, sex, lactate, pH, and prior cardiopulmonary resuscitation (CPR). Renal dysfunction (RD*crea*) was defined as median baseline creatinine ≤1.7 mg/dl vs. >1.7 mg/dl. CI, confidence interval; HFrEF, heart failure with reduced ejection fraction; HFpEF, heart failure with preserved ejection fraction; OR, odds ratio; SCAI CS class, Society for Cardiovascular Angiography & Intervention cardiogenic shock class.

***Supplementary Table 4:*** *Impact of de novo versus acute-on-chronic heart failure-related cardiogenic shock in patients with renal dysfunction.*

| **Subgroup** | **Model** | **HR (95% CI)** | **p-value** | **Interaction p-value** |
| --- | --- | --- | --- | --- |
| **de novo** | Unadjusted | 2.08 (1.54, 2.82) | <0.001 | 0.32 |
| **acute-on-chronic** |  | 1.70 (1.30, 2.22) | <0.001 |  |
| **de novo** | Model 1 | 1.80 (1.33, 2.45) | <0.001 | 0.40 |
| **acute-on-chronic** |  | 1.52 (1.16, 1.98) | 0.002 |  |
| **de novo** | Model 2 | 1.58 (1.14, 2.19) | 0.006 | 0.42 |
| **acute-on-chronic** |  | 1.33 (0.99, 1.77) | 0.056 |  |

Cohort stratified Cox proportional hazard regression models with interaction terms are shown, unadjusted, Model 1 (adjusted by age and sex), and Model 2 (adjusted by age, sex, lactate, pH, and prior CPR). Renal dysfunction (RD*gfr*) defined as median baseline cut-off value (> vs. ≤), eGFR calculated by the 2021 CKD-EPI equation. CI, confidence interval; HR, hazard ratio.

***Supplementary Table 5:*** *Association between in-hospital complications, RDcrea and AKIgfr*

| Complications | Definition | Odds Ratio (95% CI) | | P value |
| --- | --- | --- | --- | --- |
| ****Bleeding complications**** |  | |  |  |
| Moderate bleeding | RDcrea | | 1.57 (1.17, 2.12) | 0.003 |
|  | AKIgfr | | 1.11 (0.73, 1.69) | 0.62 |
| Severe bleeding | RDcrea | | 1.34 (0.9, 1.99) | 0.15 |
|  | AKIgfr | | 1.46 (0.85, 2.48) | 0.17 |
| Intervention due to bleeding | RDcrea | | 0.92 (0.49, 1.74) | 0.80 |
|  | AKIgfr | | 1.58 (0.87, 2.87) | 0.13 |
| Intracerebral bleeding | RDcrea | | 1.94 (0.82, 4.61) | 0.13 |
|  | AKIgfr | | 0.96 (0.29, 3.20) | 0.95 |
| Haemorrhagic stroke | RDcrea | | 2.17 (0.37, 12.78) | 0.39 |
|  | AKIgfr | | - | - |
| Haemolysis | RDcrea | | 1.52 (0.82, 2.78) | 0.18 |
|  | AKIgfr | | 0.51 (0.23, 1.13) | 0.10 |
| ****Ischaemic complications**** |  | |  |  |
| Ischaemic stroke | RDcrea | | 1.32 (0.75, 2.33) | 0.33 |
|  | AKIgfr | | 1.52 (0.73, 3.17) | 0.27 |
| Intervention due to access site-related ischaemia | RDcrea | | 1.36 (0.63, 2.92) | 0.43 |
|  | AKIgfr | | 1.09 (0.44, 2.72) | 0.85 |
| Laparotomy due to abdominal compartment or bowel ischaemia | RDcrea | | 1.19 (0.5, 2.83) | 0.70 |
|  | AKIgfr | | 0.73 (0.24, 2.29) | 0.59 |
| ****Other complications**** |  | |  |  |
| Hypoxic brain damage | RDcrea | | 1.06 (0.61, 1.84) | 0.83 |
|  | AKIgfr | | 1.43 (0.70, 2.92) | 0.32 |
| Renal replacement therapy | RDcrea | | 4.61 (3.28, 6.49) | <0.001 |
|  | AKIgfr | | 0.96 (0.66, 1.41) | 0.83 |
| Sepsis | RDcrea | | 2.11 (1.42, 3.13) | <0.001 |
|  | AKIgfr | | 1.03 (0.65, 1.65) | 0.89 |
| Pulmonary edema | RDcrea | | 1.08 (0.82, 1.43) | 0.58 |
|  | AKIgfr | | 0.85 (0.56, 1.29) | 0.44 |

Odds ratio calculated by mixed effects logistic regressions, adjusted by age, sex, lactate, pH, and prior cardiopulmonary resuscitation. Definitions of renal dysfunction (RD*gfr*) and acute kidney injury (AKI*gfr*) are provided in ***Supplementary Table 1***. CI, confidence interval; OR: odds ratio.

***Supplementary Table 6:*** Association between renal dysfunction, acute kidney injury, and selected treatments modalities.

| ****Category**** | ****Model**** | ****OR (95% CI)**** | ****p-value**** |
| --- | --- | --- | --- |
| Use of Vasopressors | | | |
| RD*crea* (≤ vs. >) | Unadjusted | 1.58 (1.07, 2.33) | 0.022 |
|  | Model 1 | 1.74 (1.16, 2.61) | 0.008 |
|  | Model 2 | 1.51 (0.87, 2.61) | 0.14 |
| ****AKI*crea* (Stage 0 vs. 1/2/3)**** | Unadjusted | 2.41 (1.55, 3.75) | <0.001 |
|  | Model 1 | 2.50 (1.60, 3.91) | <0.001 |
|  | Model 2 | 3.08 (1.66, 5.71) | <0.001 |

| ****MCS overall**** | | | |
| --- | --- | --- | --- |
| RD*crea* (≤ vs. >) | Unadjusted | 1.17 (0.89, 1.55) | 0.26 |
|  | Model 1 | 1.36 (1.00, 1.86) | 0.051 |
|  | Model 2 | 1.28 (0.88, 1.88) | 0.20 |
| ****AKI*crea* (Stage 0 vs. 1/2/3)**** | Unadjusted | 2.76 (2.06, 3.70) | <0.001 |
|  | Model 1 | 2.87 (2.11, 3.90) | <0.001 |
|  | Model 2 | 2.70 (1.79, 4.06) | <0.001 |
| ****Only VA-ECMO**** | | | |
| RD*crea* (≤ vs. >) | Unadjusted | 1.41 (1.01, 1.98) | 0.045 |
|  | Model 1 | 1.58 (1.10, 2.28) | 0.014 |
|  | Model 2 | 1.49 (0.92, 2.42) | 0.11 |
| ****AKI*crea* (Stage 0 vs. 1/2/3)**** | Unadjusted | 1.56 (1.09, 2.25) | 0.016 |
|  | Model 1 | 1.49 (1.03, 2.17) | 0.035 |
|  | Model 2 | 1.27 (0.78, 2.06) | 0.33 |
| ****Impella + VA-ECMO**** | | | |
| RD*crea* (≤ vs. >) | Unadjusted | 1.19 (0.76, 1.86) | 0.44 |
|  | Model 1 | 1.39 (0.86, 2.24) | 0.18 |
|  | Model 2 | 1.60 (0.86, 3.00) | 0.14 |
| ****AKI*crea* (Stage 0 vs. 1/2/3)**** | Unadjusted | 3.54 (2.14, 5.84) | <0.001 |
|  | Model 1 | 3.58 (2.15, 5.95) | <0.001 |
|  | Model 2 | 2.74 (1.43, 5.24) | 0.002 |
| ****Only Impella**** | | | |
| RD*crea* (≤ vs. >) | Unadjusted | 0.78 (0.54, 1.13) | 0.19 |
|  | Model 1 | 0.79 (0.53, 1.17) | 0.24 |
|  | Model 2 | 0.74 (0.45, 1.21) | 0.23 |
| ****AKI*crea* (Stage 0 vs. 1/2/3)**** | Unadjusted | 1.57 (1.02, 2.42) | 0.040 |
|  | Model 1 | 1.59 (1.03, 2.45) | 0.036 |
|  | Model 2 | 2.03 (1.17, 3.55) | 0.012 |

Odds ratio calculated by mixed effects logistic regressions, unadjusted, adjusted in model 1 by age and sex, and in model 2 by age, sex, lactate, pH, and prior cardiopulmonary resuscitation. Definitions of renal dysfunction (RD*crea*) and acute kidney injury (AKI*crea)* are provided in ***Supplementary Table 1***. CI, confidence interval; OR: odds ratio; VA-ECMO, veno-arterial extracorporeal membrane oxygenation.

***Supplementary Figures:***

***Supplementary Figure 1:***

*
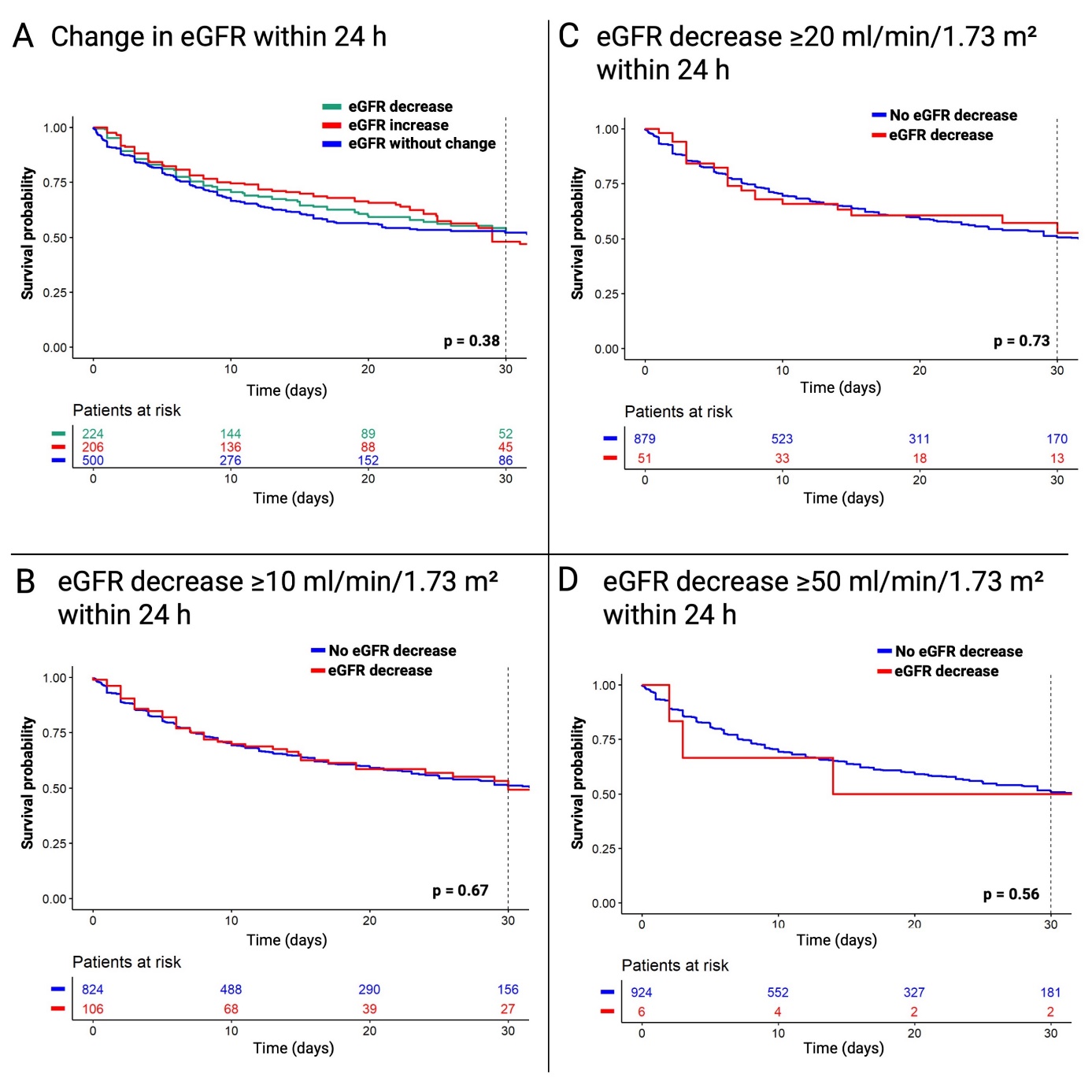
*

**Kaplan-Meier estimates for 30-day all-cause mortality in patients with heart failure-related cardiogenic shock, with verses without eGFR decrease within 24 h.** eGFR calculated using the 2021 CKD-EPI equation. Any change in eGFR within 24 h (**A**), eGFR decrease of ≥10 ml/min/1.73 m^2^ (**B**), ≥20 ml/min/1.73 m^2^ (**C**), and ≥50 ml/min/1.73 m^2^ (**D**) within 24 h.
